# Polyhexanide anti-infective coating of central venous catheters in prevention of catheter colonization and bloodstream infection: Study HC-G-H-0507

**DOI:** 10.1186/cc9649

**Published:** 2011-03-11

**Authors:** I Krikava, M Kolar, B Garajova, T Balik, A Sevcikova, J Pachl, P Sevcik, R Trubac

**Affiliations:** 1 University Hospital Brno, Czech Republic; 2 University Hospital Kralovske Vinohrady, Prague, Czech Republic; 3 B. Braun Medical s.r.o., Prague, Czech Republic

## Introduction

Internal and external anti-infective coating of central venous catheters (CVCs) may reduce the rate of catheter colonization (CC) and bloodstream infection (BSI) [1]. Our objective was to evaluate the efficacy of a protective nonleaching polyhexanide coating on the rate of CC and BSI in ICU settings.

## Methods

A prospective, randomized, controlled, double-blind clinical trial was performed on multidisciplinary ICUs of two university hospitals in the Czech Republic between 2005 and 2010. A total of 680 patients were randomized to receive either coated CVC (Certofix^® ^protect; B. Braun Melsungen AG) or standard CVC (Certofix^®^; B. Braun Melsungen AG). Primary objectives were the difference of the incidence of both CC and BSI between groups. Catheter colonization was defined as the growth of >1,000 colony-forming units using the sonication method.

## Results

**Figure 1:**
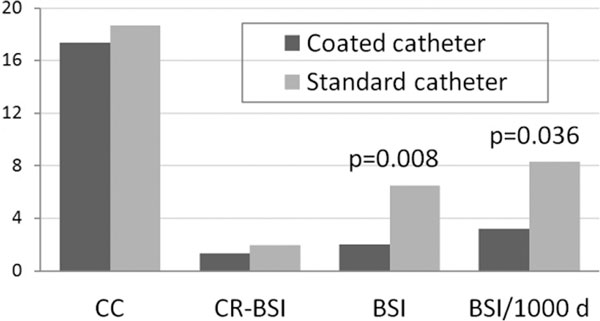


A total of 674 catheters were evaluated among which 58 catheters were excluded due to short indwelling time <3 days (an exclusion criterion). The two groups were similar with respect for the insertion site, place of insertion (ICU or surgical theatre), indwelling time, ICU stay and demographic indices. The coated CVC displayed similar incidence of CC as the standard CVCs (17.36% vs. 18.67%, *P *= 0.747) as well as incidence of catheter-related BSI (1.33% vs. 1.94%, *P *= 0.752). The rate of BSI was significantly lower in protected CVCs (2.00% vs. 6.47%, *P *= 0.008), and the incidence of BSI/1,000 catheter-days was lower in coated catheters (3.21 vs. 8.30, *P *= 0.036) as well (Figure 1).

## Conclusions

Our results suggest that the use of external/internal polyhexanide-coated CVCs is associated with significant reduction of BSI but not with the reduction of colonization rate.
